# Species check-list for Tintinnids of the Philippines Archipelago (Protozoa, Ciliophora)

**DOI:** 10.3897/zookeys.771.24806

**Published:** 2018-07-05

**Authors:** Jane Abigail Santiago, Maria Carmen Lagman

**Affiliations:** 1 De La Salle University, Taft Avenue, Manila

**Keywords:** Ciliates, list, Manila Bay, Philippine Sea, plankton, zooplankton

## Abstract

Tintinnids are an essential link between nano- and macro- planktons in the food webs of the marine environment. It is also known that tintinnids are one of themajor components of marine planktonic ciliates and has a cosmopolitan character. In the Philippine archipelago, which is recognized as a center of marine biodiversity, tintinnids checklist has not been done or published. Therefore, a checklist is presented in this study based on a compilation of previous tintinnids studies conducted at the Philippines waters. As a result of the studies done since 1941 up to present, a total of 114 taxa belonging to 14 families and 37 genera were listed. The Philippines coastal waters record a total of 50 species while the open seas document 72 species to date.

## Introduction

Microzooplankton (20–200µm) constitute amajor component of the marine plankton community. Previously, the significance of microzooplankton (MZP) was commonly linked with microbial loop and corresponding microbial web (Calbet and Landry 2004, Calbet et al. 2008), but recent studies have shown that they also play a key role in the herbivorous food web (Dolan et al. 2007, Putland and Iverson 2007). MZP graze a wide variety of particles from bacteria to nano- and phytoplankton as well as other similar organisms. They have a crucial role in the first feeding of the larval fishes (Stoecker and Capuzzo 1990, Fukami et al. 1999) and thus should be valued in the aquaculture industry. The awareness of the dynamic role of MZP in marine ecosystem resulted in the increase of scientific interest in the factors affecting their abundance and distribution. Research on microzooplankton arises as one of the vital parts of biological oceanography. In order to fully understand MZP behavior in different environments, a systematic qualitative study that includes listing of the species in a region is an essential step in exploring these organisms.

One of the best-known groups of marine microzooplanktonic ciliates is tintinnid (Kato and Taniguchi 1993). The distinctive characteristic of the tintinnid is its lorica, which has been the basis of their identification and classification. The easiness in identifying tintinnids based on their morphological features made them model specimens for research on species distributions, diversity, and variations in the structure of microzooplankton communities (Dolan and Gallegos 2001). Studies about the tintinnids distribution are essential due to the fact that they have been used as bio-indicators of different water massess (Kim et al. 2012). For example, the tintinnid species named *Epiplocyloides reticulata* (Ostenfeld & Schmidt, 1901) has been acknowledged as the Kuroshio water current indicator (Lee and Kim 2010). Records of *E. reticulata* are important to know the geographic extension of the warm Kuroshio current and the possible areas it can affect. A documentation of the tintinnid distribution is recognized as one of the best method to trace the flow of the water mass in open oceans and coastal waters (Lee and Kim 2010). In an archipelagic country such as Philippines, conducting tintinnid studies can be helpful in tracing different water masses and can aid in the assessment and management of its marine environment. However, tintinnids are poorly studied in the Philippines, a place which has been recognized as the center of the center of marine shore fish biodiversity (Carpenter and Springer 2005). A species- checklist for tintinnids specific for the Philippines can be a good starting point for any researcher who wants to conduct a tintinnid survey or any type of investigation in the country.. In order to assist other possible and future tintinnids studies in the Philippines, this present work aims to present the first and current checklist of tintinnid species in the Philippines. The authors also made this list to encourage other researcher to increase tintinnid studies in the Philippines. This study is based on a compilation of the literature to date.

## Materials and methods

The Philippines archipelago is bound by the Bashi Channel to the north, the Philippine Sea to the east and northeast, the Celebes Sea to the south, the Sulu Sea to the southwest, and the South China Sea to the west and northwest side.

In this study, all published literature from 1941 to 2017 was examined. Taxonomical species and author names were written according to Roxas (1941), Gómez (2007), Kim et al. (2012) and Santiago et al. (2017).The study of Taniguchi (1977) was not included as a reference in enumerating tintinnid species since he only referred tintinnids as a group and his paper does not contain any detailed list of tintinnid species. The WoRMS (World Register of Marine Species) data system (Warren 2018) was used for classification and basis of the current species name. The species checklist in this study is alphabetically ordered.

## Results

In related studies conducted in the Philippines, 114 tintinnid species belonging to 14 families and 37 genera have been recorded. The families Codonellidae (22 species, 19.30%) and Tintinnidae (21 species, 18.42%) have the highest recorded species (Table 3).

**Figure 1.**
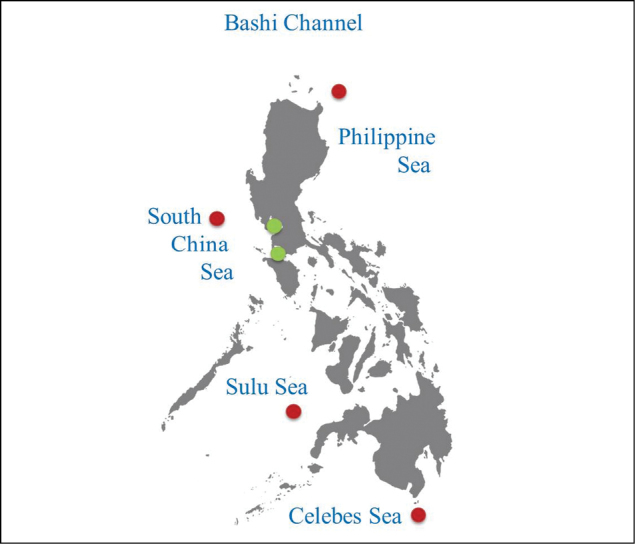
Map of the Philippines. Dots imdicate the sites with recorded tintinnid species. Key: green dots: coastal water; red dots: open sea.)

The systematic list and biogeographical distribution of the species are presented below:

**Kingdom: Chromista**

Subkingdom: Harosa

Phylum: Ciliophora Doflein, 1901

Class: Oligotrichea Bütschli, 1887

Subclass: Oligotrichia Bütschli, 1887

Order: Choreotrichida Small & Lynn, 1985

Family: **Ascampbelliellidae** Corliss, 1960

Genus: *Acanthostomella* Jörgensen, 1927

*Acanthostomella conicoides* Kofoid & Campbell, 1929

*Acanthostomella minutissima* Kofoid & Campbell, 1929

Genus: *Ascampbelliella* Corliss, 1960

*Ascampbelliella acuta* (Kofoid & Campbell, 1929)

*Ascampbelliella armilla* (Kofoid & Campbell, 1929)

*Ascampbelliella retusa* (Hada, 1935)

*Ascampbelliella urceolata* (Ostenfeld, 1899)

Genus: Craterella Kofoid & Campbell, 1929

*Craterella aperta* Marshall

Family: **Codonellidae** Kent, 1881

Genus: *Codonaria* Kofoid & Campbell, 1939

*Codonaria oceanica* (Brandt, 1906)

Genus: *Codonella* Haeckel, 1873

*Codonella amphorella* Biedermann, 1893

Genus: *Poroecus* Cleve, 1902

*Poroecus annulatus* Kofoid & Campbell, 1929

*Poroecus apicatus* Kofoid & Campbell, 1929

Genus: *Tintinnopsis* Stein, 1867

*Tintinnopsis bacoornensis* Roxas, 1941

*Tintinnopsis beroidea* Stein, 1867

*Tintinnopsis buetschlii* Daday, 1887

*Tintinnopsis campanula* Ehrenberg, 1840

*Tintinnopsis chinglanensis* Nie & Cheng, 1947

*Tintinnopsis corniger* Hada, 1964

*Tintinnopsis cylindrica* Daday, 1887

*Tintinnopsis directa* Hada, 1932

*Tintinnopsis gracilis* Kofoid & Campbell, 1929

*Tintinnopsis loricata* Brandt, 1906

*Tintinnopsis major* Meunier, 1910

*Tintinnopsis manilensis* Roxas, 1941

*Tintinnopsis mortensenii* Schmidt, 1902

*Tintinnopsis radix* (Imhof, 1886)

*Tintinnopsis rotundata* Kofoid & Campbell, 1929

*Tintinnopsis tocantinensis* Kofoid & Campbell, 1929

*Tintinnopsis turgida* Kofoid & Campbell, 1929

*Tintinnopsis uruguayensis* Balech, 1948

Family: **Codonellopsidae** Kofoid & Campbell, 1929

Genus: *Codonellopsis* Jörgensen, 1924

*Codonellopsis morchella* (Cleve) Jörgensen, 1924

*Codonellopsis orthoceras* (Haeckel, 1873) Jörgensen, 1924

*Codonellopsis ostenfeldi* (Schmidt, 1902) Kofoid & Campbell, 1929

*Codonellopsis pusilla* (Cleve) Jörgensen, 1924

*Codonellopsis schabi* (Brandt, 1906) Kofoid & Campbell, 1929

Family: **Cyttarocylididae** Kofoid & Campbell, 1939

Genus: *Cyttarocylis* Fol, 1881

*Cyttarocylis cassis* (Haeckel, 1837)

Family: **Dictyocystidae** Haeckel, 1873

Genus: Wangiella Nie, 1934

*Wangiella dicollaria* Nie, 1934

Genus: *Dictyocysta* Ehrenberg, 1854

*Dictyocysta elegans* Ehrenberg, 1854

*Dictyocysta mitra* Haeckel, 1873

Family: **Epiplocylididae** Kofoid & Campbell, 1939

Genus: *Epiplocylis* Jörgensen, 1924

*Epiplocylis calyx* (Brandt, 1906)

*Epiplocylis exquisita* Kofoid & Campbell, 1929

*Epiplocylis undella* (Ostenfeld & Schmidt) Jörgensen, 1927

Genus: *Epiplocyloides* Hada, 1938

*Epiplocyloides acuta* (Kofoid & Campbell,1929)

*Epiplocyloides ralumensis* (Brandt, 1906)

*Epiplocyloides reticulata* (Ostenfeld & Schmidt, 1901)

Family: **Metacylididae** Kofoid & Campbell, 1929

Genus: *Coxliella* Brandt

*Coxliella longa* Kofoid & Campbell, 1929

*Coxliella mariana* Hada, 1938

Genus: *Metacylis* Jörgensen, 1924

*Metacylis hemisphaerica* Roxas, 1941

*Metacylis jörgensenii* (Cleve) Kofoid & Campbell, 1929

*Metacylis kofoidi* Roxas, 1941

*Metacylis tropica* Duran, 1957

Genus: *Helicostomella* Jörgensen, 1924

*Helicostomella longa* (Brandt, 1906)

Genus: *Climacocylis* Jörgensen, 1924

*Climacocylis elongata* Kofoid & Campbell, 1929

Climacocylis cf. leospiralis Kofoid & Campbell

*Climacocylis scalaria* Brandt, 1906

*Climacocylis sipho* (Brandt, 1906) Kofoid & Campbell, 1929

Family: **Petalotrichidae** Kofoid & Campbell, 1929

Genus: *Petalotricha* Kent, 1881

*Petalotricha major* Jörgensen, 1925

Family: **Ptychocylididae** Kofoid & Campbell, 1929

Genus: *Favella* Jörgensen, 1924

*Favella ehrenbergii* (Claparède & Lachmann, 1858) Jörgensen, 1924

*Favella simplex* Roxas, 1941

*Favella philippinensis* Roxas, 1941

*Favella elongata* Roxas, 1941

*Favella azorica* (Cleve, 1900) Jörgensen, 1924

Family: **Rhabdonellidae** Kofoid & Campbell, 1929

Genus: *Rhabdonella* Brandt, 1906

*Rhabdonella amor* (Cleve, 1900) Brandt, 1907

*Rhabdonella apophysata* Jörgensen, 1924

*Rhabdonella brandti* Kofoid & Campbell, 1929

*Rhabdonella conica* Kofoid & Campbell, 1929

*Rhabdonella cornucopia* Kofoid & Campbell, 1929

*Rhabdonella elegans* Jörgensen, 1924

*Rhabdonella exilis* Kofoid & Campbell, 1929

*Rhabdonella sanyahensis* Nie & Cheng, 1947

*Rhabdonella fenestrata* Roxas, 1941

*Rhabdonella valdestriata* (Brandt) Kofoid & Campbell, 1929

*Rhabdonella spiralis* (Fol, 1881)

Genus: *Protorhabdonella* Jörgensen, 1924

*Protorhabdonella curta* Cleve, 1900

*Protorhabdonella simplex* (Cleve) Jörgensen, 1924

*Protorhabdonella striatura* Kofoid & Campbell, 1929

Family: **Tintinnidae** Claparède & Lachmann, 1858

Genus: *Amphorellopsis* Kofoid & Campbell, 1929

*Amphorellopsis acuta* (Schmidt, 1902)

Genus: *Amphorides* Strand, 1928

*Amphorides amphora* (Claparède & Lachmann, 1858)

*Amphorides quadrilineata* (Claparède & Lachmann, 1858)

*Amphorides minor* Jörgensen, 1924

Genus: *Brandtiella* Kofoid & Campbell, 1929

*Brandtiella palliata* (Brandt, 1906) Kofoid & Campbell, 1929

Genus: *Canthariella* (Kofoid & Campbell, 1929)

*Canthariella pyramidata* (Jörgensen, 1924) Kofoid & Campbell, 1929

Genus: *Dadayiella* Kofoid & Campbell, 1929

*Dadayiella ganymedes* (Entz, 1884) Kofoid & Campbell, 1929

*Dadayiella pachytoecus* (Dendy, 1924)

Genus: *Eutintinnus* Kofoid & Campbell, 1939

*Eutintinnus apertus* Kofoid & Campbell, 1929

*Eutintinnus fraknoii* (Daday, 1887)

*Eutintinnus lusus-undae* (Entz, 1885)

*Eutintinnus stramentus* (Kofoid & Campbell, 1929)

Genus *Ormosella* Kofoid & Campbell, 1929

*Ormosella haeckeli* Kofoid & Campbell, 1929

Genus: *Salpingella* Jörgensen, 1924

*Salpingella acuminata* (Claparède & Lachmann, 1858) Jörgensen, 1924

*Salpingella acuminatoides* (Laackmann) Kofoid & Campbell, 1929

*Salpingella attenuata* Kofoid & Campbell, 1929

*Salpingella decurtata* Jörgensen, 1924

*Salpingella subconica* Kofoid & Campbell, 1929

Genus: *Steenstrupiella* Kofoid & Campbell, 1929

*Steenstrupiella intumescens* (Jörgensen, 1924) Kofoid & Campbell, 1929

*Steenstrupiella steenstrupii* (Claparède & Lachmann, 1858) Kofoid & Campbell, 1929

Genus: *Tintinnus* Schrank, 1803

*Tintinnus perminutus* Kofoid & Campbell, 1929

Family: **Tintinnidiidae** Kofoid & Campbell

Genus: *Tintinnidium* Kent, 1881

*Tintinnidium primitivum* Busch, 1923

*Tintinnidium cylindrica* Daday, 1886

*Tintinnidium ampullarium* Roxas, 1941

Genus: *Leprotintinnus* Jörgensen, 1899

*Leprotintinnus nordqvistii* (Brandt, 1906) Kofoid & Campbell, 1929

*Leprotintinnus tubulosus* Roxas, 1941

Family: **Undellidae** Kofoid & Campbell, 1929

Genus: *Undella* Daday, 1887

*Undella claparedei* (Entz) Daday, 1887

*Undella clevei* Jörgensen, 1924

*Undella hyalina* Daday, 1887

*Undella subcaudata* Jörgensen, 1924

Family: **Xystonellidae** Kofoid & Campbell, 1929

Genus: *Parundella* Jörgensen, 1924

*Parundella aculeata* (Joergensen, 1924)

*Parundella caudata* (Ostenfeld, 1899) Jörgensen, 1924

*Parundella inflata* Kofoid & Campbell, 1929

*Parundella longa* Joergensen, 1924

Genus: *Xystonella* Brandt, 1907

*Xystonella treforti* (Daday, 1887)

Genus: *Xystonellopsis* Jörgensen, 1924

*Xystonellopsis brandti* (Laackmann) Jörgensen, 1924

*Xystonellopsis cymatica* (Brandt, 1906) Jörgensen, 1924

*Xystonellopsis dahli* (Brandt, 1906) Kofoid & Campbell, 1929

*Xystonellopsis paradoxa* (Cleve, 1900) Jörgensen, 1924

The study of Roxas (1941) contained the first recorded tintinnid species in the Philippines. Roxas (1941) documented 32 tintinnid species wherein ten were newly discovered species (Table 2). *Favella simplex*, *Favella philippinensis*, and *Favella elongata* were the only accepted and registered species in the WoRMS database (Warren 2018) among the said newly discovered species. The other newly discovered species are still included in this present checklist due to the scarcity of tintinnid studies in the Philippines. The other newly discovered species were not recorded in any other studies and we took into consideration that they might be endemic in the area where Roxas (1941) collected them. Roxas also misspelled *Leprotinntinnus nordqvistii*, which he recorded as *Leprotinntinnus nordquisti*.

Since 1941, only three other studies (Gómez 2007, Kim et al. 2012, Santiago et al. 2017) were made in the Philippines that identified tintinnids to species level. The paper of Roxas (1941) and Santiago et al. (2017) recorded a total of 50 tintinnid species from coastal waters of Manila Bay (39 species) and Puerto Galera Bay (11 species). On the other hand, Gómez (2007) and Kim et al. (2012) conducted their sample collection within the Philippines open seas, which amounted to 72 tintinnid species.

*Tintinnopsis, Codonellopsis, Coxliella, Metacylis, Rhabdonella, Epiplocylis* and *Eutintinus* were the genera that both appeared in coastal and open waters (Table 1). There were eight genera that were only recorded in coastal waters and a total of 24 genera were solely found in the open seas (Table 1). *Epiplocylis undella* and *Rhabdonella spiralis* were the only species common to all of four tintinnid studies in the Philippines (Table 2).

**Table 1.** Summary of the tintinnid appearance between coastal and open seas by genus.

**Total**	**Coastal**	**Open sea**	**Both**
	*Favella*	*Acanthostomella*	*Codonellopsis*
	*Helicostomella*	*Amphorellopsis*	*Coxliella*
	*Leprotintinnus*	*Amphorides*	*Epiplocylis*
	*Petalotricha*	*Ascampbelliella*	*Eutintinnus*
	*Tintinnidium*	*Brandtiella*	*Metacylis*
	*Tintinnus*	*Canthariella*	*Rhabdonella*
	*Wangiella*	*Climacocylis*	*Tintinnopsis*
		*Codonaria*	
		*Codonella*	
		*Craterella*	
		*Cyttarocylis*	
		*Dadayiella*	
		*Dictyocysta*	
		*Epiplocylididae*	
		*Epiplocyloides*	
		*Ormosella*	
		*Parundella*	
		*Poroecus*	
		*Protorhabdonella*	
		*Salpingella*	
		*Steenstrupiella*	
		*Undella*	
		*Xystonella*	
		*Xystonellopsis*	
	**7**	**24**	**7**

**Table 2.** Distribution of tintinnid species reported in the Philippines. The open sea has records from the southwest (**SW**) seas that include Sulu, Celebes and South China Sea (Gómez 2007). The northeast (**NE**) was based on the study of Kim et al. (2012) in the Philippine Sea. The species in the Coastal areas were from Manila bay (**MB**) (Roxas 1941, Santiago et al. 2017) and Puerto Galera Bay (**PG**) (Roxas 1941). An asterisk (*) denotes new species.

Taxon	Open sea		Coastal	
	SW	NE	MB	PG
1. *Acanthostomella conicoides*		+		
2.*Acanthostomella minutissima*	+			
3. *Amphorellopsis acuta*		+		
4. *Amphorides amphora*	+	+		
5. *Amphorides minor*		+		
6. *Amphorides quadrilineata*	+	+		
7. *Ascampbelliella acuta*		+		
8. *Ascampbelliella armilla*	+			
9. *Ascampbelliella retusa*	+			
10. *Ascampbelliella urceolata*		+		
11. *Brandtiella palliata*	+	+		
12. *Canthariella pyramidata*	+	+		
13. *Climacocylis cf. leospiralis*	+			
14. *Climacocylis elongata*		+		
15. *Climacocylis scalaria*	+	+		
16. *Climacocylis sipho*		+		
17. *Codonaria oceanica*	+			
18. *Codonella amphorella*		+		
19. *Codonellopsis morchella*			+	
20. *Codonellopsis orthoceras*		+	+	
21. *Codonellopsis ostenfeldi*			+	
22. *Codonellopsis pusilla*	+			
23. *Codonellopsis schabi*	+			
24. *Coxliella longa*			+	
25. *Coxliella mariana*		+		
26. *Craterella aperta*	+			
27. *Cyttarocylis cassis*		+		
28. *Dadayiella ganymedes*	+	+		
29. *Dadayiella pachytoecus*		+		
30. *Dictyocysta elegans*	+	+		
31. *Dictyocysta mitra*		+		
32. *Epiplocylis calyx*		+		
33. *Epiplocylis exquisita*				+
34. *Epiplocylis undella*	+	+	+	+
35. *Epiplocyloides acuta*		+		
36. *Epiplocyloides ralumensis*				+
37. *Epiplocyloides reticulata*	+			
38. *Eutintinnus apertus*	+			
39. *Eutintinnus fraknoii*	+	+	+	
40. *Eutintinnus lusus-undae*	+	+	+	
41. *Eutintinnus stramentus*	+	+		
42. *Favella azorica*				+
43. *Favella ehrenbergii*			+	
44. Favella elongate*			+	
45. Favella philippinensis*			+	
46. Favella simplex*			+	
47. *Helicostomella longa*			+	
48. *Leprotintinnus nordqvistii*			+	
49. Leprotintinnus tubulosus*			+	
50. Metacylis hemisphaerica*				+
51. *Metacylis jörgensenii*		+	+	
52. Metacylis kofoidi*				+
53. *Metacylis tropica*			+	
54. *Ormosella haeckeli*		+		
55. *Parundella aculeata*	+			
56. *Parundella caudata*		+		
57. *Parundella inflata*		+		
58. *Parundella longa*	+			
59. *Petalotricha major*				+
60. *Poroecus annulatus*	+			
61. *Poroecus apicatus*		+		
62. *Protorhabdonella curta*	+	+		
63. *Protorhabdonella simplex*	+	+		
64. *Protorhabdonella striatura*		+		
65. *Rhabdonella amor*	+	+		+
66. *Rhabdonella apophysata*	+	+		
67. *Rhabdonella brandti*				+
68. *Rhabdonella conica*			+	
69. *Rhabdonella cornucopia*		+		
70. *Rhabdonella elegans*	+			
71. *Rhabdonella exilis*		+		
72. Rhabdonella fenestrata*				+
73. *Rhabdonella sanyahensis*				
74. *Rhabdonella spiralis*	+	+	+	+
75. *Rhabdonella valdestriata*		+		
76. *Salpingella acuminata*	+	+		
77. *Salpingella acuminatoides*		+		
78. *Salpingella attenuata*	+			
79. *Salpingella decurtata*	+			
80. *Salpingella subconica*		+		
81. *Steenstrupiella intumescens*		+		
82. *Steenstrupiella steenstrupii*	+	+		
83. Tintinnidium ampullarium*			+	
84. *Tintinnidium cylindrica*			+	
85. *Tintinnidium primitivum*			+	
86. Tintinnopsis bacoornensis*			+	
87. *Tintinnopsis beroidea*			+	
88. *Tintinnopsis buetschlii*			+	
89. *Tintinnopsis campanula*	+			
90. *Tintinnopsis chinglanensis*			+	
91. *Tintinnopsis corniger*			+	
92. *Tintinnopsis cylindrica*			+	
93. *Tintinnopsis directa*			+	
94. *Tintinnopsis gracilis*			+	
95. *Tintinnopsis loricata*			+	
96. *Tintinnopsis major*			+	
97. Tintinnopsis manilensis*			+	
98. *Tintinnopsis mortensenii*			+	
99. *Tintinnopsis radix*			+	
100. *Tintinnopsis rotundata*			+	
101. *Tintinnopsis tocantinensis*			+	
102. *Tintinnopsis turgida*			+	
103. *Tintinnopsis uruguayensis*			+	
104. *Tintinnus perminutus*			+	
105. *Undella claparedei*	+	+	+	
106. *Undella clevei*	+			
107. *Undella hyalina*		+		
108. *Undella subcaudata*	+			
109. *Wangiella dicollaria*			+	
110. *Xystonella treforti*	+	+		
111. *Xystonellopsis brandti*		+		
112. *Xystonellopsis cymatica*	+	+		
113. *Xystonellopsis dahli*		+		
114. *Xystonellopsis paradoxa*		+		
108. *Undella subcaudata*	+			
109. *Wangiella dicollaria*			+	
110. *Xystonella treforti*	+	+		
111. *Xystonellopsis brandti*		+		
112. *Xystonellopsis cymatica*	+	+		
113. *Xystonellopsis dahli*		+		
114. *Xystonellopsis paradoxa*		+		
108. *Undella subcaudata*	+			
109. *Wangiella dicollaria*			+	
110. *Xystonella treforti*	+	+		
111. *Xystonellopsis brandti*		+		
112. *Xystonellopsis cymatica*	+	+		
113. *Xystonellopsis dahli*		+		
114. *Xystonellopsis paradoxa*		+		

**Table 3.** Percentage (%) Distribution of Tintinnids families from the Philippines.

Family	Genus	Species	%
Ascampbelliellidae	3	7	6.14
**Codonellidae**	**4**	**22**	**19.30**
Codonellopsidae	1	5	4.39
Cyttarocylididae	1	1	0.88
Dictyocystidae	2	3	2.63
Epiplocylididae	2	6	5.26
Metacylididae	4	11	9.65
Petalotrichidae	1	1	0.88
Ptychocylididae	1	5	4.39
Rhabdonellidae	2	14	12.28
**Tintinnidae**	**10**	**21**	**18.42**
Tintinnidiidae	2	5	4.39
Undellidae	1	4	3.51
Xystonellidae	3	9	7.89

## Discussion

Presently, there are only four related studies (Roxas 1941, Gómez 2007, Kim et al. 2012, Santiago et al. 2017) that contain tintinnid species in the Philippines. Roxas (1941) and Santiago et al. (2017) conducted their zooplankton collection within the Philippines coastal waters while Gómez (2007) and Kim et al. (2012) had cruises along the open seas. Table 1 and 2 showed the tintinnids distribution between open seas and coastal waters. This is an important data because some of the tintinnids were categorized into biogeographical groups (Pierce and Turner 1993). The studies (Lee and Kim 2010, Kim et al. 2012) that utilized tintinnids as indicator species used their biogeographical groups to assess water quality and mass movements. In this present study, there are species and genera that were only recorded in one area and some both appeared in open seas and coastal waters. Hence, the variation of the tintinnids distribution between open seas and coastal waters in this current work might help in further classification of tintinnid species to their biogeographical groups.

It should also be noted that each of the said four studies had a different sampling technique and effort. Roxas (1941) towed a no. 20 plankton net with 176 mesh per inch which means that it has an aperture of 0.076 mm or 76 µm. The plankton net that Santiago et al. 2017 used has 64 µm mesh size. These can indicate that the majority of the collected species of Roxas (1941) and Santiago et al. 2017 were large tintinnid species (>64 µm). Microzooplankton size range from 20 to 200 µm, thus, collecting tintinnids through plankton net with a relatively larger aperture size can result in loss of most of the smaller-sized tintinnids.

In the studies conducted in Philippines open seas, Gómez (2007) used Niskin bottles while Kim et al. (2012) towed a 20 µm mesh-plankton-net. The differences in methodologies and lack of standardization of sampling technique on tintinnids collection (Gómez 2007) can add complication on the analysis and comparison of their biogeographical distribution. Apparently, more studies on tintinnids in the Philippines and a standard of methodology should be established. The authors executed this current work to serve as a starting point for other researchers and encourage them to conduct studies on tintinnids in a center of marine biodiversity such as the Philippines.
